# Estrogen receptor α in cancer associated fibroblasts suppresses prostate cancer invasion *via* reducing CCL5, IL6 and macrophage infiltration in the tumor microenvironment

**DOI:** 10.1186/s12943-015-0488-9

**Published:** 2016-01-20

**Authors:** Chiuan-Ren Yeh, Spencer Slavin, Jun Da, Iawen Hsu, Jie Luo, Guang-Qian Xiao, Jie Ding, Fu-Ju Chou, Shuyuan Yeh

**Affiliations:** George Whipple Lab for Cancer Research, Departments of Urology and Pathology, University of Rochester Medical Center, Rochester, NY 14642 USA; Department of Pathology, University of Rochester Medical Center, Rochester, NY 14642 USA

**Keywords:** CAF, ERα, CCL5, IL6, Tumor associated macrophages, Prostate cancer

## Abstract

**Background:**

Cancer associated fibroblasts (CAF) play important roles in tumor growth that involves inflammation and epithelial cell differentiation. Early studies suggested that estrogen receptor alpha (ERα) was expressed in stromal cells in normal prostates and prostate cancer (PCa), but the detailed functions of stromal ERα in the PCa remain to be further elucidated.

**Methods:**

Migration and invasion assays demonstrated the presence of high levels of ERα in CAF cells (CAF.ERα(+)) suppressed PCa invasion *via* influencing the infiltration of tumor associated macrophages. ERα decreased CAF CCL5 secretion *via* suppressing the CCL5 promoter activity was examined by luciferase assay. ERα decreased CCL5 and IL-6 expression in conditioned media that was collected from CAF cell only or CAF cell co-cultured with macrophages as measured by ELISA assay.

**Results:**

Both in vitro and in vivo studies demonstrated CAF.ERα(+) led to a reduced macrophage migration toward PCa *via* inhibiting CAF cells secreted chemokine CCL5. This CAF.ERα(+) suppressed macrophage infiltration affected the neighboring PCa cells invasion and the reduced invasiveness of PCa cells are at least partly due to reduced IL6 expression in the macrophages and CAF.

**Conclusion:**

Our data suggest that CAF ERα could be applied as a prognostic marker to predict cancer progression, and targeting CCL5 and IL6 may be applied as an alternative therapeutic approach to reduce M2 type macrophages and PCa invasion in PCa patients with low or little ERα expression in CAF cells.

**Electronic supplementary material:**

The online version of this article (doi:10.1186/s12943-015-0488-9) contains supplementary material, which is available to authorized users.

## Background

Prostate cancer (PCa) is the most frequently diagnosed cancer and second leading cause of cancer death in men in the United States [1]. PCa is a chronic type of tumor that requires a long time for small lesions to become clinically manifested compared to some other cancers [2]. Inflammation has been thought to be one of the key pathogenic factors for PCa and there is an association between chronic inflammation and increased prevalence of PCa [3–6]. Furthermore, tumor associated macrophages (TAM) form a major component of the inflammatory infiltrates in both primary and secondary tumors [7] and can release growth factors, cytokines and chemokines to regulate tumor growth and invasion [8]. However, the detailed mechanisms how the interactions among stromal cells, TAM, and PCa cells could influence the growth and metastasis of PCa remain to be further elucidated.

An earlier study suggested that cancer associated fibroblasts (CAF) may play important roles to influence PCa progression and invasion [9]. In the prostate tumor microenvironment (TME), PCa epithelial cells can produce some growth factors, such as TGF-β, PDGF and FGF, to influence/activate peripheral stromal cells that result in transformation of normal fibroblasts into CAF. Subsequently, CAF can then increase in population through transforming from normal fibroblasts [10], differentiation from bone marrow-derived mesenchymal stem cells [11] or by epithelial to mesenchymal transition (EMT). The important functions of CAF include the regulation of deposition of extracellular matrix (ECM), epithelial differentiation, tumor inflammation, and wound healing [12]. Ezer et al. demonstrated that CAF could mediate inflammation and angiogenesis by recruiting macrophages to stimulate angiogenesis, which may then promote tumor growth [13].

The existence of aromatase (to convert testosterone to estrogen) [14] and the finding of an increase in estrogen-to-androgen ratio in aging men [15] indicated that estrogens, in addition to androgens, could play important roles in PCa initiation and progression. Animal studies also demonstrated that 100 % of rats being treated with 17β-estradiol (E_2_) plus testosterone for around 44 weeks had prostatic adenocarcinomas [16].

Estrogen action is mainly mediated through its specific nuclear receptors that regulate transcription of target genes *via* binding to the estrogen response element (ERE) or non-ERE mediated transactivation, as well as non-genomic regulations [17]. There are two major types of estrogen receptors (ERs), ER alpha (ERα) and ER beta (ERβ) [18, 19]. The two ER subtypes are structurally similar, consisting of the six common domains (A–F), but encoded by separate genes (*ESR1 and ESR2*). Immunostaining indicated that ERα positive [ERα(+)] staining was present in normal prostate stromal cells nuclei [20]. The function of stromal ERα, however, remains largely unknown.

It has been well demonstrated that cancer related inflammation promotes cancer cells proliferation, migration and invasion through several pathways, including signal transduction activation, cytokines secretion and immune cells infiltration [21]. The TAM, M2 type, are the major players that link tumor related inflammation and tumor progression [22]. A variety of chemokines, like CCL2 and CCL5, have been detected in neoplastic tissues and associated with tumor associated immune cells formation and recruitment [23].

Using the in vitro co-culture system and in vivo mouse models, we studied CAF ERα roles in PCa invasion and found CAF ERα could inhibit PCa metastasis *via* suppression of macrophage infiltration and M2 type macrophages formation. This CAF.ERα(+) → macrophages → PCa invasion pathway involves the modulation of CAF CCL5 and macrophages IL6 gene expressions. This finding supports the clinical observation that PCa patients with stromal ERα have better PSA free survival rates [24].

## Results

### ERα in CAFs suppressed macrophage infiltration

**Fig. 1:**
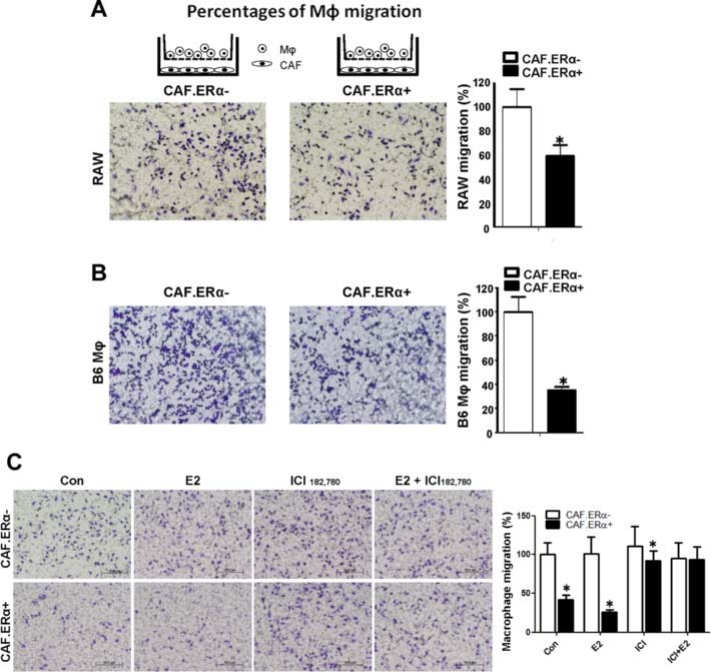
ERα expression in CAF reduced the macrophage (Mφ) migration. **a** and **b** CAF ERα reduces the migration of macrophages. CAF.ERα(+) or ERα(−) cells were cultured in 24-well plates for 24 h. We then added macrophages in inserted upper transwells (5 μm pore size) for 24 h and then compared macrophages migration rate toward CAF.ERα(−) vs. CAF.ERα(+) cells. **c** 17β-estradiol (E2) treatment can reduce macrophage migration in CAF.ERα(+) cells and ICI182,780 (ICI) treatment can reverse E2 effects. CAF.ERα(−) or CAF.ERα(+) cells were first cultured in media with 5 % CD FBS for 2 days, then seeded in 24-well plates, and incubated with vehicle, E2 (10 nM) or/and ICI (10 μM) for 24 h. We then added macrophages into inserted upper transwells (5 μm pore size) for 24 h and compared macrophage migration rates toward CAF.ERα(−) vs. CAF.ERα(+). Migrated macrophages were fixed and stained by 1 % toluidine blue in PBS. Quantifications are in right panels. *, *P* < 0.05 vs. CAF.ERα(−) cells

Early reports showed that ERα in stromal cells could affect the prostate development [25, 26]. Another report showed that E2 plus testosterone treatment could stimulate the PCa initiation [27], however, the role of stromal ERα in the later stages of PCa progression and how it may affect immune cell infiltration and PCa metastasis is not well studied. Although the positive expression of ERα in the CAF is lower than in the benign component of human PCa tissues [28], the clinical correlation has been identified and one study showed patients with CAF.ERα(+) expression have better PSA free recurrence survival rate [24]. We isolated CAF from TRAMP mice prostate tumors, immortalized them by SV40 large T-antigen, and then studied how ERα in CAF cells may affect the infiltrating macrophages. Using a transwell system of adding macrophage RAW-264.7 cells on the insert wells and seeding CAF.ERα(+) or CAF.ERα(−) cells in the bottom chambers, we found chambers seeded with CAF.ERα(+) had less macrophages infiltrated than with CAF.ERα(−) cells (Fig. 1a). Similar results were obtained when we replaced mouse macrophage RAW-264.7 cells with B6 mouse primary macrophages (Mφ) (Fig. 1b). We also compared macrophages recruitment between CAF.ERα(−) and CAF.ERα(+) with/without E2 treatment. Our results indicated that E2 treatment can further reduce CAF.ERα(+) diminished macrophage recruitment and treatment with ICI182,780 can reverse E2 and ERα reduced macrophage infiltration (Fig. 1c).

Together, results from Fig. 1 suggest that CAF with ERα expression could reduce macrophage population in the PCa microenvironment.

### Infiltrating macrophages enhance PCa invasion

**Fig. 2:**
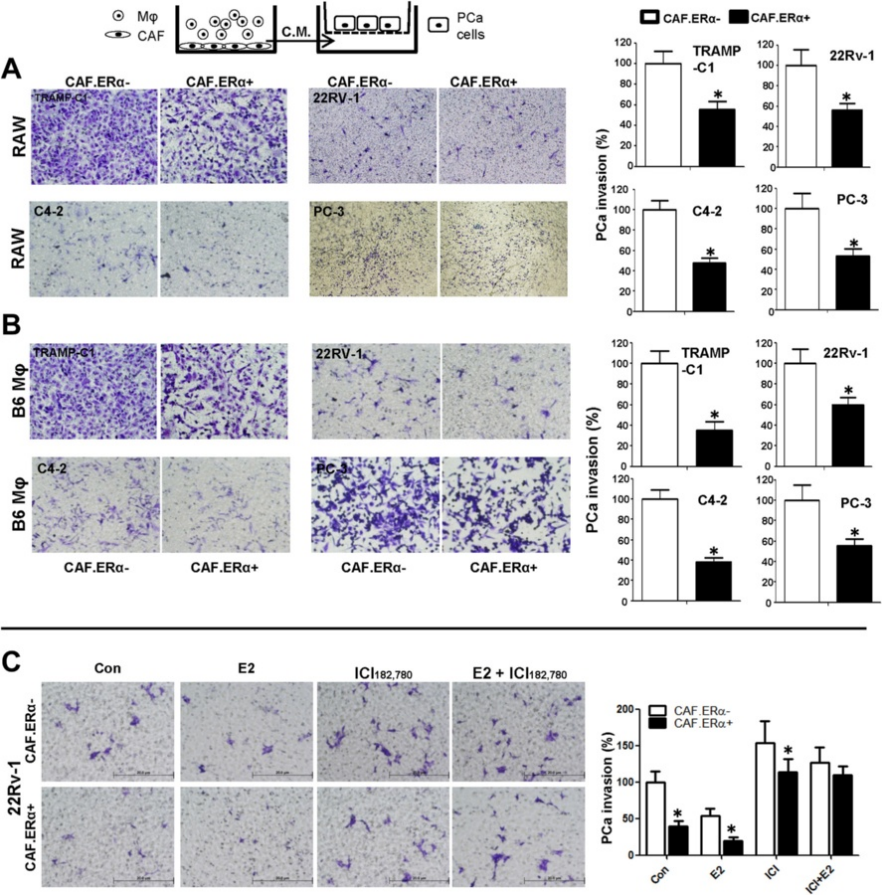
Effects of CM from co-cultured CAF.ERα(+)/macrophages or CAF.ERα(−) /macrophages (Mφ) on PCa invasion. The carton illustrates the PCa invasion transwell system. CM was collected from 48 h co-culture of CAF.ERα(+) or CAF.ERα(−) and RAW264.7 (RAW) cells (**a**) or B6 primary macrophages (Mφ) (**b**), co-cultured CM was added to 24-well plates and the PCa cells TRAMP-C1, CWR22Rv-1 (22Rv1), C4-2, or PC-3, were seeded into inserted transwells pre-coated with matrigel. After 24 to 48 h incubation (TRAMPC-1 and PC-3 for 24 h; CWR22Rv-1 and C4-2 for 48 h), invaded PCa cells were counted and compared between CM of CAF.ERα(−)/macrophages and CAF.ERα(+)/macrophages. **c** Estrogen treatment further triggers CAF.ERα(+) reduced PCa invasion. CAF.ERα(−) or ERα(+) cells were treated with vehicle, E2 (10 nM) or/and ICI (10 μM) and co-cultured with macrophages for 48 h. CMs were collected and added to 24-well plates and the PCa cells (CWR22Rv-1) were seeded onto inserted transwells pre-coated with matrigel. After 48 h incubation, invaded PCa cells were counted and compared, and quantitation data is shown at right. *, *P* < 0.05 vs. CAF.ERα(−)/macrophages CM treatment group

To further study the consequences of altered infiltrating macrophages on PCa invasion, we co-cultured mouse CAF.ERα(+) or CAF.ERα(−) cells with mouse macrophages and then collected the conditioned media (CM) to assay the influence on the invasiveness of mouse PCa cells (TRAMP-C1). As shown in Fig. 2a, the CM from co-culture of CAF.ERα(+) and mouse RAW264.7 cells led a lower TRAMP-C1 cells invasion as compared to CM from co-culture of CAF.ERα(−) and RAW-264.7 cells. Similar results were obtained when we replaced TRAMP-C1 cells with human PCa cells CWR22Rv-1 (22Rv1), C4-2, or PC-3 cells (Fig. 2a). Furthermore, replacing mouse macrophage RAW-264.7 cells with B6 primary macrophages (Mφ) also showed similar results (Fig. 2b). We also evaluated ERα activity by E2 and ICI182,780 to confirm stromal ERα role in PCa invasion. We co-cultured CAF.ERα(−)/macrophages, CAF.ERα(+)/macrophages with/without ICI182,780 and/or E2 treatment for 2 days. CMs were collected to induce CWR22Rv-1 cells invasion. Our data showed E2 treatment can suppress PCa invasion but adding ICI 182,780 can partially reverse this decreased invasion (Fig. 2c). We also observed similar results in C4-2 cells (Additional file 1: Figure S1).

To mimic the in vivo tumor micro-environment, we applied the 3D invasion assay system to confirm the outcomes from the transwell invasion assay. In the 3D assay, the formation of acini-like structures counts as an indicator for invasion [29]. CM collected from CAF.ERα(−)/macrophages co-culture increased the CWR22Rv-1 cell formation of acini-like structures as compared to CM from CAF.ERα(+)/macrophages co-culture (Additional file 2: Figure S2A). We also found decreased laminin 5 (an indicator of increased invasion) in PCa cells cultured with the CM from the CAF.ERα(−)/macrophages as compared to CM from CAF.ERα(+)/macrophages (Additional file 2: Figure S2B). Importantly, the expression of the key invasion marker, MMP9, was 2 fold higher in CWR22Rv1 cells treated with CM of CAF.ERα(−)/RAW264.7 co-culture than those treated with CM from CAF.ERα(+)/RAW264.7 co-culture (Additional file 2: Figure S2C) in this 3D invasion system.

Together, results from Fig. 2 and Additional file 2: Figure S2 using different invasion assays with different macrophages and PCa cells all suggested that ERα in CAF could suppress PCa invasion at least partly *via* suppressing the infiltrating macrophages.

### CAF.ERα(+) suppresses PCa invasion *via* reduced macrophage infiltration in the in vivo mouse PCa model

**Fig. 3:**
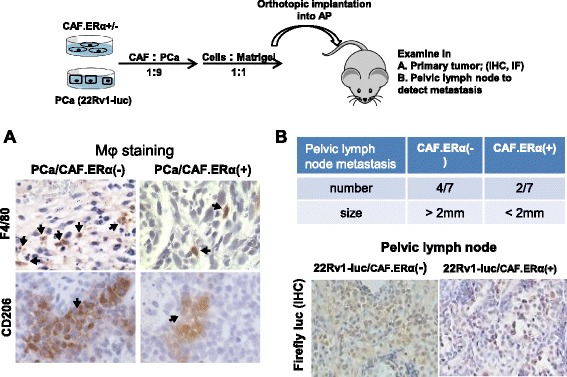
In vivo tumor model demonstrated CAF ERα reduced macrophages infiltration and PCa cell invasion. Nude mice were orthotopically implanted with CAF.ERα(+) or CAF.ERα(−) mixed with CWR22Rv1 cells that were stably transfected with firefly luciferase (22Rv1-Luc). **a** Tumors were collected 12 weeks after implantation, and macrophage infiltration was examined by IHC staining of F4/80 (M1 macrophage, upper panel) and CD206 (M2 macrophage, lower panel) [30]. **b** Pelvic lymph nodes were collected from CAF.ERα(−)/22Rv1-Luc and CAF.ERα(+)/22Rv1-Luc co-cultured groups to determine metastasis by measuring sizes of lymph nodes. We confirmed, by IHC staining of luciferase, the presence of 22Rv1-Luc cells in the lymph nodes (lower panel). There were seven mice in each group. We defined pelvic lymph nodes as metastatic when the diameter was over 2 mm (upper panel) *, *P* < 0.05 *vs*. CAF.ERα(−)/22Rv1-Luc tumors

To confirm the above in vitro results in the in vivo animal model, we orthotopically co-implanted CAF.ERα(+) or CAF.ERα(−) plus CWR22Rv-1 cells. CWR22Rv1 cells were stably transfected with firefly luciferase (22Rv1-Luc) to monitor tumor implantation, growth, and metastasis using the non-invasive in vivo IVIS imaging system. Twelve weeks after implantation, tumors were collected from both primary and metastatic sites (Fig. 3). We compared the infiltrated macrophages by IHC staining and found less infiltrated macrophages, including M1 (F4/80) and M2 (CD206) macrophages [30], in CAF.ERα(+)/22Rv1-Luc primary tumors than in the CAF.ERα(−)/22Rv1-Luc primary tumors (Fig. 3a). We carefully examined the tumor histology, and found that in the co-implants of CAF.ERα(−) and PCa cells, our data show tumors were big, cells were more dense, and necrosis could be observed in the central part of tumor (data not shown). We also found that 4 out of 7 CAF.ERα(−)/22Rv-1-Luc co-implanted mice and 2 out of 7 CAF.ERα(+)/22Rv-1-Luc co-implanted mice with enlarged pelvic lymph nodes, but the numbers of enlarged pelvic lymph nodes were variable in individual mice. Among those mice positive for enlarged lymph nodes, the CAF.ERα(−) implanted mice presented larger pelvic lymph nodes than the CAF.ERα(+) implanted group. The length or width of nodes in CAF.ERα(−) mice were ≥2 mm, but not in those CAF.ERα(+) implanted group. Thus, we used 2 mm width or length to define the malignancy of this metastatic PCa. To verify the enlarged pelvic lymph nodes were correlated to metastasis, but not inflammation, the pelvic lymph nodes were IHC stained for presence of luciferase. Importantly, this luciferase staining found fewer metastatic tumors in pelvic lymph nodes in mice co-implanted with CAF.ERα(+)/22Rv1.Luc cells (Fig. 3b).

Together, results from Fig. 3 suggest that CAF.ERα(+) may suppress PCa invasion at least partly *via* altering the macrophage infiltration into PCa in the in vivo mouse model.

### Mechanism dissection how CAF ERα(+) suppresses macrophage infiltration

**Fig. 4:**
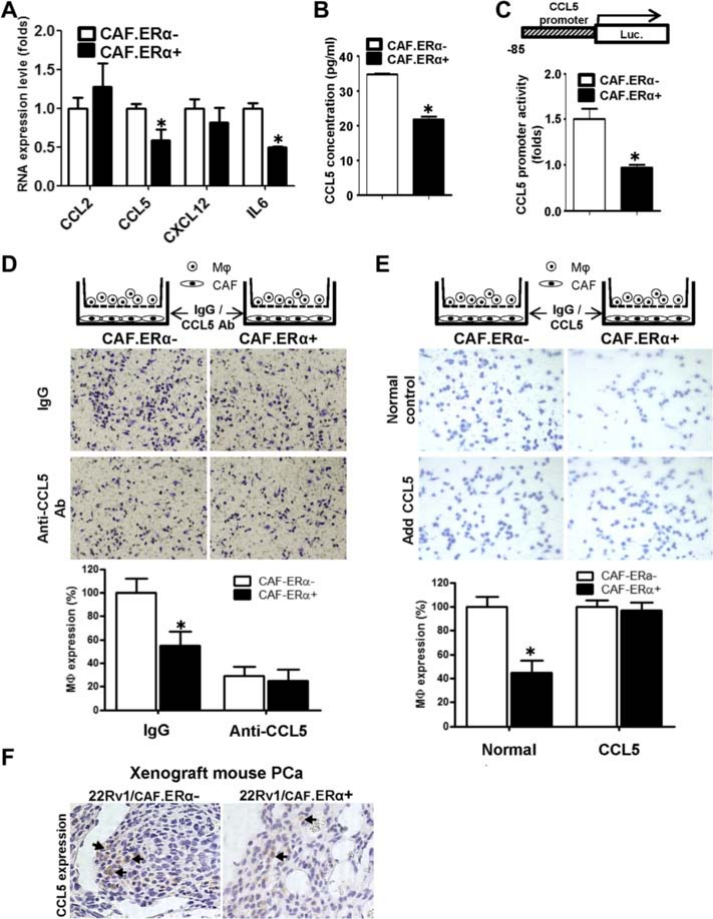
ERα reduced the CCL5 expression in CAF cells and consequently decreased  macrophages infiltration. **a** We compared gene profiles of macrophage attraction related chemokines/cytokines between CAF.ERα(+) and CAF.ERα(−) by QPCR. **b** The comparison of CCL5 production from CAF.ERα(+) and CAF.ERα(−) was determined by ELISA through measuring CCL5 concentration in the CM. **c** CCL5 promoter luciferase activity was used to determine whether ERα regulates CCL5 expression. CCL5 promoter (−83 bp)-luciferase reporter was transfected into CAF.ERα(+) and CAF.ERα(−) and cultured in CD-FBS media. 10 nM E2 was added and CCL5 promoter luciferase activity was analyzed using a dual luciferase kit. **d** CAF were cultured in bottom wells and incubated with CCL5 neutralizing Ab or IgG (control). After 24 h, macrophages were added into inserted upper transwells that also contained CCL5 neutralizing Ab or IgG (control). Migrated macrophages were counted and compared to CAF.ERα(−) with IgG as control. Quantification is in lower panel. **e** Adding recombinant CCL5 protein reversed CAF.ER(+) reduced macrophage migration. CAF cells were incubated with recombinant CCL5 protein or control for 24 h, and then macrophages with recombinant CCL5 protein or control were added to the inserted transwells for migration assay. All migrated macrophages were compared to CAF.ERα(−) with control protein. Quantification is in lower panel. **f** CCL5 expression was confirmed by IHC staining in the in vivo mouse PCa tumors co-implanted with/without both CAF/22Rv1-Luc cells. *Arrows* show positive CCL5 staining. *, *P* < 0.05 *vs*. CAF.ERα(−)/22Rv1 tumors

To dissect the molecular mechanism(s) by which CAF.ERα(+) expression could suppress macrophage infiltration, we performed Q-PCR gene expression assays with several macrophage migration-related chemokines and cytokines, including the family of the C-C motif chemokine ligand (CCL) and interleukin (IL) genes. Our results showed that CCL5 and IL6 gene expression were significantly decreased in CAF.ERα(+) cells as compared to those found in CAF.ERα(−) cells (Fig. 4a). We also co-cultured macrophages with CAFs to study whether macrophages can impact ERα functions in CAF cells. The results showed that compared to CAFs alone, the co-culture of macrophages with CAFs resulted in increased CCL5 and IL6 expression in CAF cells. Consistently, higher ERα expression in CAF decreased CCL5 and IL6 in CAF cells while comparing CAF.ERα(+)/macrophages vs. CAF.ERα(−)/macrophages group (Additional file 3: Figure S3). The IL6 neutralizing antibody did not affect the CAF regulated macrophage migration/infiltration (data not shown). To further confirm CCL5 expression was altered at the protein level, we assayed CCL5 concentration in culture media using ELISA, and results indicated that secreted CCL5 protein decreased in CAF.ERα(+) media (Fig. 4b).

There are two identified transcription factor binding sites in the CCL5 promoter region, including binding sites for NF-κB (−70 to −58) and (−55 to −42), and a SP1/KLF binding site (−70 to −58). Previous studies showed that through a non-ERE pathway the ERα could regulate downstream genes activities, including NF-κB and AP-1 [31, 32]. We therefore focused on examining whether ERα could modulate the CCL5 at the transcriptional level by characterizing the CCL5 promoter (−83 bp) that was constructed into a luciferase reporter [33]. Our results showed that CCL5 luciferase activity is higher in CAF.ERα(−) than in CAF.ERα(+) cells (Fig. 4c).

We also applied an interruption approach to test if interrupting the CCL5 signal with CCL5 neutralizing antibody may block the effects of CAF.ERα(−) on macrophages infiltration in the co-culture system. Our results showed the CCL5 neutralizing antibody could significantly and effectively diminish the CAF.ERα(−) modulated macrophage infiltration toward CAF with less effect on CAF.ER(+) modulated infiltration (Fig. 4d, quantification in lower panel).

Then, we applied another interruption approach *via* adding CCL5 recombinant protein to examine whether the ectopic CCL5 could restore/reverse the CAF.ERα(+) cells' low-capacity to recruit macrophages. Indeed, our results showed adding CCL5 protein could increase the low-capability of CAF.ERα(+) cells to attract macrophages, indicating the lower CCL5 is a key factor that leads to lower macrophages attraction of CAF.ERα(+) (Fig. 4e). We then examined the CCL5 expression in mice with in vivo co-implanted CWR22Rv1-Luc cells with CAF with or without ERα (PCa:CAF = 9:1) and IHC staining of CCL5 data showed less CCL5 positive signals in CAF.ERα(+) than in CAF.ERα(−) implanted group (Fig. 4f).

Together, results from Fig. 4a–f suggest that CAF.ERα(+) cells have a lower ability to attract macrophages due to a lower chemokine CCL5 expression.

### Mechanisms of CAF.ERα + suppressed PCa invasion

**Fig. 5:**
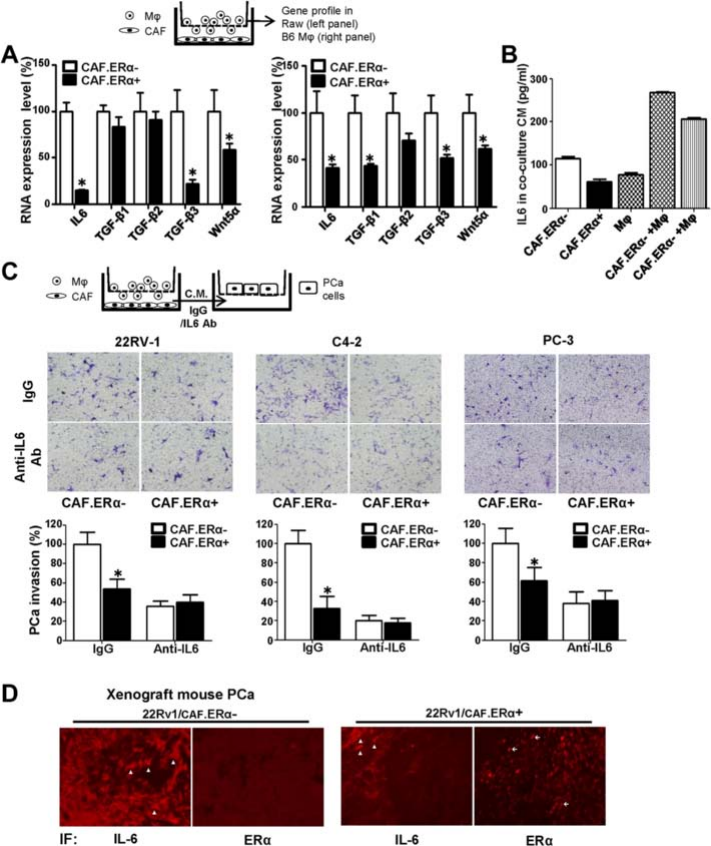
CAF.ERα(+) CM treated macrophages have a reduced capability of producing IL6, which could consequently reduce PCa invasion. **a** We compared metastatic-related gene profile expressions by QPCR in macrophages after co-culture with CAF.ERα(+) or CAF.ERα(−) cells. Macrophages were seeded in bottom wells, then CAF.ERα(+) or CAF.ERα(−) cells were seeded onto inserted transwells (0.4 μm) and co-cultured for 24 h. Macrophage RNA was collected and converted to cDNA. Selected metastatic related genes expressions in macrophage were measured by QPCR, RAW cells in left panel and Mφ in right panel. **b** IL6 concentration in CM from CAF/macrophage co-inoculation was measured by ELISA. **c** IL6 neutralizing antibody blocks macrophages promoted PCa invasion. The next experiment compared macrophages that were incubated with CM either from CAF.ERα(+) or CAF.ERα(−). PCa cells (CWR22Rv1, C4-2, or PC3) were seeded onto matrigel pre-coated transwells for 48 h to demonstrate invasive ability. **d** There is a lower IL6 staining in CAF.ERα(+)/CWR22Rv1 co-implanted tumors. *Arrowheads* show the cells that express IL6 protein. *Arrows* indicate cells positive for the ERα expression. IL6 expression is reversely correlative to CAF ERα expression using IF staining. *, *P* < 0.05 *vs*. CAF.ERα-/ 22Rv1 tumors

Next, we examined the molecular mechanism(s) by which the CAF.ERα(+) affected macrophages could influence PCa cell invasion. As shown in Fig. 5a, we compared gene profiles of both RAW-264.7 and B6 primary macrophages co-cultured with CAF.ERα(+) or CAF.ERα(−) cells, and found lower IL6 expression in both types of macrophages after co-culture with CAF.ERα(+) cells. Although TGF-β3 and Wnt 5α expressions increase, yet their mRNA amounts and expression levels are not as high as IL6. We therefore set the priority to focus on studying IL6. We further examined the altered IL6 expression at the protein level *via* ELISA assay and data showed there is less IL6 in the CM from CAF.ERα(+)/macrophages co-culture system. This indicates that CAF.ERα(+) cells have less capability to stimulate IL-6 secretion from macrophage RAW-264.7 cells (Fig. 5b). As expected, adding IL6 neutralizing antibody into the CM diminished the CAF.ERα(−)/macrophage mediated PCa invasion of CWR22Rv-1, C4-2 and PC3 cells (Fig. 5c). We further demonstrated CAF.ERα signals-mediated CAF IL-6 reduction could also impact PCa invasion. We put CM that was collected from CAF.ERα(−) or CAF.ERα(+) cells with neutralizing IL6 antibody or IgG (control) into bottom wells, and seeded CWR22Rv1 or C4-2 cells into matrigel-coated transwell for invasion assay. Our results showed that IL6 neutralizing antibody can decrease CAF-induced PCa cell invasion (Additional file 4: Figure S4). In vivo evidence from orthotopically xenografted mouse PCa also confirmed the above in vitro data showing CAF.ERα(+)/22Rv-1 tumors have less IL6 expression (Fig. 5d). We also confirmed IL6 and ERα correlation by IF staining of serial section slides. Our results further indicated there was little IL-6 positive signal when stromal ERα is positively expressed.

Together, results from Fig. 5a–d and Additional file 4: Figure S4 suggest that CAF.ERα(+) may be able to lower the production of IL6 in macrophages, consequently reduce the macrophages-mediated PCa cell invasion.

### Correlative expression of ERα, M2 macrophage, CCL5 and IL6 in human prostate tumor

**Fig. 6:**
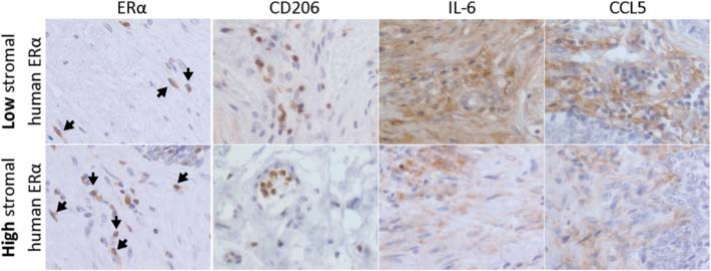
M2 macrophages, CCL5 and IL6 expression increase in prostate cancer patients with high stromal ERα expression. To confirm our findings in human prostate tumors, we also examined ERα, CD206 (M2 macrophage marker), CCL5 and IL6 in human samples by IHC staining. Samples were provides by Department of Pathology, University of Rochester Medical center

To confirm our findings in human prostate tumors, we further examined the ERα, CD206, CCL5 and IL6 expressions in 14 human PCa tissue specimens by IHC staining. Our results showed a positive correlation between ERα, M2 macrophages, CCL5 and IL-6. In high stromal ERα expression samples, the expression levels of CD206 (M2 macrophage marker), CCL5 and IL6 were higher than in samples with low stromal ERα expression (Fig. 6). These results are consistent with our in vitro and in vivo studies.

## Discussion

In the TME, chronic inflammation has been proven to promote cancer progression [34]. Tumor cells can secrete chemokines, cytokines and prostaglandins for inflammatory cells recruitment in order to sustain the inflammatory response. Nelson et al. [35] indicated that inflammation plays an important role in the development and progression of PCa. The chronic inflammation mainly occurred in the area directly adjacent to PCa lesions and induced inflammatory cell infiltration/accumulation. After immune cells accumulated at the sites, the tumor consequently increased prostate epithelial cells proliferation by inflammatory oxidants secretion [36]. Furthermore, cancer related inflammation may affect tumor cell migration, invasion, angiogenesis, etc. Not only epithelial cells, but also CAF  can produce inflammatory factors and affect immune cells recruitment. Among several chemokines, CCL1, −2, −4, −5, −7, −8, −12, −13, and IL6 might influence the interaction of inflammation with cancer malignancy, and CCL2, −3, −5, −7, CXCL12, −14, and IL6 were found to be able to affect the macrophage infiltration [37]. In our study, we found that expression of ERα in CAF can reduce the number of infiltrated macrophages recruited by CAF and PCa cells and subsequently suppress cancer invasion.

In the cancer initiation stage, epithelial cancer cells can activate and differentiate fibroblasts into myofibroblasts and the activated fibroblasts consequently promote tumor growth [38, 39]. When tumors progress, the ratio of cancer cells to CAFs may vary depending on the stages of the disease. A previous study showed that epithelial and CAF cells were set at different ratios to study the interaction between fibroblasts and different breast cancer cells [40]. In a prostate cancer study, Camps et al. co-injected PC-3 cells with CAFs (PCa:CAF = 10:1; 1 × 10^6^:1 × 10^5^) into mice and successfully promoted tumor growth [41]. In another of our studies, we co-injected CWR22Rv-1 cells and CAFs (22Rv-1:CAFs = 9:1; 9 × 10^5^:1 × 10^5^) into each lobe of mouse anterior prostates and tested whether the ERα status in CAFs could promote or inhibit tumor invasion [24]. When we changed the PCa:CAF ratio from 9:1 to 5:1, we could also see the similar effects (data not shown). The data presented in this study was collected from PCa:CAF at ratio 9:1. In addition to determining the CAF.ERα-regulated PCa invasion, in another of our projects studying CAF ERα role in PCa growth, we found the differential roles of CAF.ERα(+). CAF cells with higher ERα expression could promote the growth, but inhibit the invasion of PC3, LNCaP, C4-2 and CWR22Rv-1 cells. The in vivo model also demonstrated mice co-injected with CWR22Rv-1 and CAF.ERα(+) cells can develop bigger tumors yet lower metastasis rates as compared to mice co-injected with CWR22Rv-1 and CAF.ERα(−) cells (Da and Yeh et al., paper in preparation).

CAF have been demonstrated to play important roles in cancer progression through promoting tumor initiation, growth and invasion *via* promotion of the extracellular matrix (ECM) remodeling and release of growth factors and cytokines. CAF are a source of ECM-degrading proteases such as the MMPs [42], which might allow cancer cells to escape the primary tumor site. Our previous study also indicated CAF.ERα(+) suppressed PCa metastasis through decreased Thbs2 and MMP3 expression [24]. Other studies demonstrated liver CAF could induce metastases through secreting inflammatory factors, like IL6 and MCP-1 [43, 44]. In addition, CAF have the capability to recruit immune cells into the tumor region *via* altering the expression of IL6, CCL2 [45], or NF-kB signals [13].

Our findings indicated CAF cells expressing ERα have a lower capability to recruit macrophages. Further mechanism dissection showed that both CCL5 and IL6 secretions are decreased in CAF.ERα(+), with CCL5 subsequently related to macrophage recruitment, but not IL6. We hypothesized that CCL5 may play a key role for recruiting the infiltrating macrophages to PCa cells. Robinson et al. also demonstrated that CCL5 plays an important role in attracting macrophage migration and may become a target for breast cancer therapy [46]. In a breast cancer murine model, those murine cells treated with Met-CCL5 (receptor antagonist) had a decreased number of infiltrating macrophages associated with a significantly reduced tumor size. The development of “anti-macrophages” may become one option for cancer therapy in the future. M2 type macrophages, one type of inflammatory cells that are differentiated by IL-4 and IL-13 stimulations, are known as major mediators linking cancer and inflammation [22, 47]. Recent data showed CAF, through stromal-derived growth factor-1 secretion, promote M2-type macrophages expression and PCa progression [48]. We examined M2 macrophages related markers expression in macrophages after CAF CM treatment. Surprising, after co-culture with the CAF.ERα(+)CM, the macrophages expressed less M2 macrophage markers, including IL-10, Fuzz1 and Ym1, but not arginase-1 (Additional file 5: Figure S5A), suggesting CAF.ERα(+) may be able to suppress M2-type macrophages in the PCa TME. This conclusion is further supported by the finding of higher IL-4 and IL-13 expression in CAF.ERα(−) than in CAF.ERα(+) cells (Additional file 5: Figure S5B). This suggests CAF.ERα(+) cells can release less IL-4 and IL-13 and may induce less M2-type macrophages than CAF.ERα(−) cells.

In prostate development, using Cre-loxP gene knockout strategy, reports have shown that ERα plays different roles in prostate epithelial as well as different types of prostate stromal cells [26, 49]. In the PCa mouse models, both ERα knockout [50] and ERα agonist treatment [51] showed mice with activated ERα can develop high-grade PIN, suggesting ERα might play important roles in PCa progression. Early studies indicated the expression of epithelial ERα, but not stromal ERα, was increased in PCa [52]. Celhay et al. demonstrated stromal ERα may also play an important role in recurrence of hormone refractory PCa. They compared ERα expression by IHC in 55 paired patient PCa samples collected before androgen deprivation therapy and after hormonal relapse. They found a shorter time to hormonal relapse was associated with low staining for ERα in stromal cells and correlated to shorter patient survival rate [53]. Daniels et al. [28] reported that ERα positive rates reduced in the cancer associated stromal cells compared to the adjacent benign prostate tissue. Although the expression level of ERα in cancer associated stromal cells is relatively weak, the intensity of ERα expression in tumor-associated stroma shows a positive correlation with cancer progression. The reduced CAF ERα IHC staining by Daniels et al. [28] supports our finding that CAF ERα plays a protective role in cancer invasion. Furthermore, PCa patients with CAF.ERα(+) expression have a better PSA free recurrence survival rate [24]. Our data demonstrated stromal ERα can inhibit PCa invasion through suppressing macrophage infiltration into tumor sites and directly decrease cytokine secretion in PCa cells.

Platz et al. indicated chronic inflammation could be an epidemiologic factor for PCa [54], and De Marzo et al. also linked the PCa progression to inflammation related dietary factors [4]. Prins et al. [55] demonstrated that estrogen induced inflammation is specifically mediated by epithelial ERα. The epithelial inflammatory cell infiltrates were observed with aging in wild type and ERβ knock out (ERβKO), but not in ERαKO, mice after DES (Diethylstilbestrol) treatment. Van Laere et al. demonstrated that activation of NF-kB in inflammatory breast cancer was associated with loss of ERα expression, suggesting ERα might play a positive role in anti-inflammation [56]. In autoimmune encephalomyelitis, ERα-ligands mediated anti-inflammation is important in neuroprotection for reducing the levels of central nervous system inflammation [57]. ERα has been proven to have an anti-inflammatory function in macrophages. However, the ERα roles in inflammation-mediated PCa progression may depend on the ERα location. Our data showed stromal ERα can decrease macrophage infiltration, but may also suppress CAF-mediated inflammation response.

Our results showed ERα in CAF not only decreases IL6 expression in CAF cells, but also regulates macrophages activity to decrease IL6 secretion, although the mechanisms by which CAF.ERα(+) cells affect macrophage activity are still unclear. Previous studies indicated IL6 and leukemia inhibitory factor (LIF) secretion increases in tumor tissues can promote TAM generation. Deprivation of IL6 and LIF can suppress TAM induction. Early studies indicated that inflammatory cytokines, such as IL6, might play major roles in the metastasis of breast and neck cancers [58, 59]. Michalaki et al. [60] measured serum IL6 concentration from patients and found it is higher in patients with metastatic disease than localized disease. Lou et al. [61] also determined IL6 plays an important role in the PCa metastatic Stat3 signaling transduction pathway. But, after CAF CM treatment, we found IL6 expression in PCa cells shows no significant difference between CAF cells with/without ERα. Hsu et al. also found anti-IL6 might suppress the MMP2 and MMP9 expressions in a colon cancer model [62]. Importantly, Karin et al. demonstrated estrogen and propyl pyrazole triol (PPT, ERα specific agonist) could suppress metastasis of hepatocellular carcinoma *via* inhibition of IL6 expression [63]. They also indicated that the gender difference in tumor susceptibility resulted from a downregulation of IL6 production by macrophages in response to estrogens.

## Conclusion

**Fig. 7:**
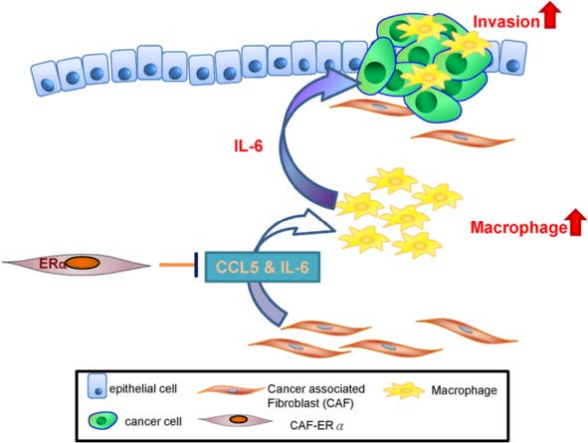
CAF.ERα(+) decreases prostate cancer invasion *via* diminishing tumor associated macrophage infiltration and IL6 secretion. Schematic diagram shows that CAF.ERα(+) cells diminishes macrophage recruited toward to PCa via reducing CAF CCL5 secretion. These CAF.ERα(+) cells reduced invasion of PCa cells are at least partly due to reduced IL6 expression in the macrophages and CAFs

Current concepts of PCa therapy mainly focus on applying anti-androgens/blocking AR activity. An increasing body of studies indicated targeting AR could suppress PCa growth but promote metastasis. Meanwhile, targeting sex hormones with various therapies may have dramatic effects, but also result in side effects. In this study, our results suggest that CAF ERα could be applied as a prognostic marker to predict cancer progression, and targeting CCL5 and IL6 may be applied as an alternative therapeutic approach to reduce M2 type macrophage and PCa invasion in CAF.ERα(−) PCa patients (Fig. 7). Our study provides candidates, like CCL5 and macrophages, for cancer therapy. In the future, we can try to block the CCL5 signaling pathway to evaluate the possibility of CCL5 in PCa treatment.

## Methods

### Cell lines

Four PCa cell lines, TRAMP-C1, CWR22Rv-1, C4-2, and PC-3, and the mouse macrophage cell line, RAW264.7, were purchased from the American Type Culture Collection (ATCC, Rockville, MD). CAF were prepared as a primary culture from 36-weeks-old TRAMP mice and immortalized by SV40 large T-antigen using the detailed process described previously [24]. All cells were maintained in RPMI media with 10 % FBS and 1 % penicillin/streptomycin.

### Isolation and primary culture of macrophages from B6 mice

B6 mice were euthanized by CO_2_ asphyxiation and cervical dislocation. After sterilization, femur bones were isolated and sterilized in 70 % ethanol and rinsed with PBS. We cut the bones at both ends, flushed the bone-marrow out with RPMI media with 10 % heat-inactivated FBS using syringes with 25-gauge needles. Bone marrow fluid was centrifuged at 1200 x rpm for 10 mins, and cells were cultured with RPMI media with macrophages colony-stimulating factor (M-CSF 20 ng/ml). After 6 days of culture, primary macrophages became mature for experimentation.

### Lentiviral ERα transduction of CAF cells and firefly luciferase transduction of CWR22Rv-1 cells

The ERα cDNA was cloned into PmeI site of pWPI lentiviral vector. The 293 T packaging cells were transiently transfected with pMD2.G and psPAX2 with pWPI-vector or pWPI-ERα, to produce lentiviral particles. The supernatants containing lentiviral particles were collected 48 h post-transfection of 293 T cells, and polybrene was added. The lentiviral supernatants were then filtered and used to transduce CAF for 48 h. The viral vector or ERα transduced CAF were then subjected to 5 mg/L blasticidin selection. To monitor tumor progression by In vivo Imaging System (IVIS) system, CWR22Rv-1 cells were tagged with firefly luciferase by lentivirus system.

### Migration assay

CAF.ERα(+) or CAF.ERα(−) were cultured in 24-well plates. After 24 h, macrophages were seeded on the inserted transwells. After 24 h co-incubation, transwells were washed with PBS and then fixed by 75 % ethanol. Next, transwell membranes were stained with 1 % toluidine blue (w/v, prepared in PBS) and non-migrated macrophages, remaining on the inner transwell surface, were wiped off. Macrophages that migrated to the bottom side of membranes were counted in ten representative areas *via* microscope (x100 fold).

#### Invasion assay

Conditioned media (CM) collected from the CAF/macrophages co-culture system were used to attract PCa cells invasion *via* matrigel coated (0.2 mg/ml, 100 μl, air dried overnight) transwells. For the co-culture system, CAF.ERα(+) or CAF.ERα(−) were seeded in the bottom wells of 6-well plates and macrophages were added into the top transwells (pore size is 0.4 μm). The CM was collected from bottom wells after 48 h co-culture. Then, CM was added into each well of new 24-well plates, then matrigel coated transwells were inserted and PCa cells (TRAMP-C1, CWR22Rv1, C4-2, or PC-3, as in figures) at 5 × 10^4^/150 μl were seeded on each transwell. After 24 h incubation, transwells were washed, fixed, and stained. The method for counting invaded cell numbers was the same as with migration assay.

#### Immunohistochemistry (IHC)

IHC staining was carried out as described previously [64]. Sections were incubated with the primary antibodies, anti-F4/80 (anti-mouse macrophages, Biolegend, San Diego, CA), anti-CD206 (anti-M2 macrophage; sc-20150, Santa Cruz, Dallas, TX), anti-CCL5 (Ameritech Biomedicines, Houston, TX), anti-IL6 (Abcam, ab6672, Cambridge, MA) and anti-firefly (Santa Cruz, Dallas, TX), in 3 % BSA in PBS overnight at 4 °C followed by respective secondary antibodies.

#### RNA extraction and quantitative real-time PCR analysis

Total RNA was extracted by Trizol reagent (Invitrogen, Carlsbad, CA) according to the manufacturer’s instructions. RNAs (1 μg) were subjected to reverse transcription using Superscript III transcriptase (Invitrogen). The obtained cDNAs were applied for qPCR using a SYBR green Bio-Rad CFX96 system. Primers used are listed in Table 1. Gene mRNA expression levels were normalized to the mRNA level of GAPDH.

**Table 1:** Sequence of qPCR primers

Name		Sequence, 5′ → 3′
*Ccl2*	sense	TAA AAA CCT GGA TCG GAA CCA AA
	antisense	GCA TTA GCT TCA GAT TTA CGG GT
*Ccl5*	sense	TAT CCT GGT GGA GTT GTG
	antisense	CAG AGT CAT CCC TGC TTC
*Cxcl-12*	sense	TGC ATC AGT GAC GGT AAA CCA
	antisense	CAC AGT TTG GAG TGT TGAG GAT
*IL6*	sense	CTG CAA GAG ACT TCC ATC CAG
	antisense	AGT GGT ATA GAC AGG TCT GTT GG
*MMP1*	sense	CCC TGG GAA GCT GTT ATC TTC AA
	antisense	CGA CCC ACT TCT GAT GGG CT
*MMP2*	sense	ACC TGA ACA CTT TCT ATG GCT G
	antisense	CTT CCG CAT GGT CTC GAT G
*MMP9*	sense	GGA CCC GAA GCG GAC ATT G
	antisense	CGT CGT CGA AAT GGG CAT CT
*MMP13*	sense	TGT TTG CAG AGC ACT ACT TGA A
	antisense	CAG TCA CCT CTAAGCC AAA GAA A
*Fizz1*	sense	CCA ATC CAG CTA ACT ATC CCT CC
	antisense	ACC CAG TAG CAG TCA TCC CA
*Arginase 1*	sense	TGT CCC TAA TGA CAG CTC CTT
	antisense	GCA TCC ACC CAA ATG ACA CAT
*Ym1*	sense	CAG GTC TGG CAA TTC TTC TGA A
	antisense	GTC TTG CTC ATG TGT GTA AGT GA
*IL4*	sense	ATC ATC GGC ATT TTG AAC GAG G
	antisense	TGC AGC TCC ATG AGA ACA CTA
*IL13*	sense	TGA GCA ACA TCA CAC AAG ACC
	antisense	GGC CTT GCG GTT ACA GAG G
*GAPDH*	sense	AAT GTC ACC GTT GTC CAG TTG
	antisense	GTG GCT GGG GCT CTA CTT C

#### CCL5 promoter luciferase assay

CCL5 promoter luciferase activity was performed using Lipofectamin 2000 (Invitrogen). CAF cells were transfected with CCL5-Luc (0.4 μg) and 1 ng pRL-TK-Luc reporter gene. After transfection, the media were refreshed to 10 % charcoal/dextran stripped (CD)-FBS media for 24 h and 10 nM E2 was added as indicated for an additional 24 h. Cells were then harvested for the dual luciferase assay kit (Promega, Madison, WI).

#### ELISA

CM was collected from CAF only or CAF co-cultured with macrophages for ELISA analyses of CCL5 and IL6 (eBioscience, San Diego, CA) according to the manufacturer’s instructions.

#### Orthotopic implantation

For the orthotopic implantation in mice, CWR22Rv-1 cells were transduced with firefly cDNA (22Rv1-Luc). CAF were mixed with 22Rv1-Luc cells (1:9 ratio) and injected into anterior prostate of 8 weeks old athymic nude mice [24, 41]. For cells injection, 22Rv1-Luc/CAF cells (9:1 ratio, total 1 × 10^6^) were suspended in 20 μl of media and Matrigel mix (1:1, v:v). Seven animals were used per group. Mice were monitored by IVIS every 2 weeks for tracking tumor growth and metastasis by intraperitoneal injection of luciferin (Gold Biotechnology, St. Louis, MO) to allow the luciferase to fluoresce. Tumors from primary and metastatic sites were collected after a final IVIS imaging at 12 weeks after implantation. Tumor sizes and macrophages infiltration were compared after IHC staining by macrophage markers. Lymph nodes were stained with firefly luciferase antibody (Santa Cruz, c-12) to confirm cancer cells metastasized from the primary tumor sites. All mice experiments were performed under a protocol approved by the Institutional Animal Care and Use Committee (IACUC) of the University of Rochester Medical Center.

#### Statistical analysis

Values were expressed as mean ± standard deviation (S.D.). The Student’s *t* test was used to calculate two-sided *P* values, and considered statistically significant when *P* < 0.05.

## Additional files

Additional file 1: Figure S1. Stromal E2/ERα signals negatively-regulate the PCa invasion. CAF.ERα(-) or ERα(+) cells were treated with vehicle, E2 (10 nM) or/and ICI_182,780_ (10 μM) and co-cultured with macrophages for 48 hr. CMs were collected and added to 24-well plates and the PCa cells (C4-2) were seeded into inserted transwells pre-coated with matrigel. After 48 hr of incubation, invaded PCa cells were counted and compared, and quantitation data is shown below the images. Additional file 2: Figure S2. CM from co-cultured CAF/macrophages affects PCa invasion in the 3D culture system. The carton illustrates the experimental system. CM was collected from co-culture of CAF.ERα(+) or CAF.ERα(-) cells and RAW264.7 cells or B6 primary macrophages (Mφ) for 24 hr. The CM was then used to treat CWR22Rv-1 cells for 3 days, then seeded in 3D environment for 12 days to form inter-acinar bridges. (A) Numbers of inter-aciniar bridges were counted per field and quantifications are in right panels. (B) Laminin 5 mRNA was quantified to show the invasive potential of the PCa cells in the 3D culture environment. At Day 12, RNA was extracted from PCa cells and expression levels of laminin 5 were measured by qPCR and quantifications are shown. (C) Expression of invasion related marker, MMP9, was also demonstrated by Q-PCR in CWR22Rv-1 cells pre-inoculated with the collected CM, quantification is shown. *, P < 0.05 vs. CM from CAF.ERα(-)/macrophage group. Additional file 3: Figure S3. Infiltrated Macrophages (Mφ) can affect the macrophages recruited-related gene profiles in CAF cells. CAF.ERα(-) or CAF.ERα(+) cells were co-cultured with macrophages for 2 days. We compared gene profiles of macrophages attraction related CCL5 and IL6 in CAF.ERα(+) and CAF.ERα(-) using qPCR. Additional file 4: Figure S4. Co-culture of CAF ERα(+) cells and PCa cells can decrease PCa cell invasion through changing IL-6 expression. CM was collected from CAF.ERα(-) and CAF.ERα(+) cells together with or without with IL-6 neutralizing antibody (Anti IL-6) or IgG (control) into bottom wells of 24-well transwell systems. We then trypsinized and seeded CWR22Rv-1 and C4-2 cells (1x10^5^) into matrigel-pre-coated transwells for invasion assay. Quantitation is at right. Additional file 5: Figure S5. CAF.ERα(+) can reduce the M2 marker expressions in the co-cultured macrophages (Mφ). (A) ERα in CAF cells inhibits M2-type macrophages transformation. (B) mRNA expressions of IL-4 and IL-13 in CAF cells were assayed by qPCR. mRNA expressions of M2 markers in macrophages were assayed by qPCR. After incubating with CM collected from CAF.ERα(+) or CAFERα(-), macrophages were collected to detect M2 markers by qPCR. *, P < 0.05 vs. CAF.ERα(-) cells; §, P < 0.05 vs. Mφ; δ, p < 0.05 vs. Mφ/CAF.ERα(-) CM.

## Abbreviations

CAF: cancer associated fibroblasts
Mφ: macrophages
TAM: tumor associated macrophages
EMT: epithelial to mesenchymal transition
TME: tumor microenvironment
