# Supplementary figures and images for: Estrogen receptor α in cancer associated fibroblasts suppresses prostate cancer invasion via reducing CCL5, IL6 and macrophage infiltration in the tumor microenvironment
Source: Mol Cancer. 2016 Jan 20;15:7. doi: 10.1186/s12943-015-0488-9 (PMC4721150; doi:10.1186/s12943-015-0488-9)

Yeh *et al.*, Supplementary Fig. 1.

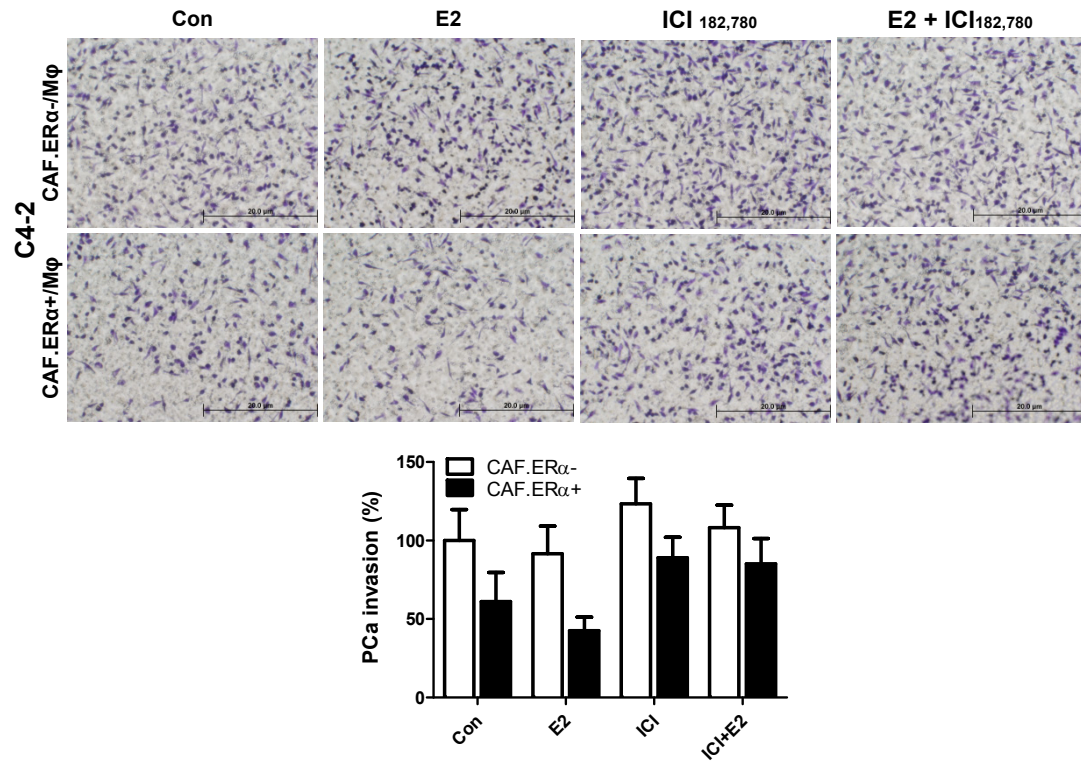

Supplement: Additional file 1: Figure S1. — Stromal E2/ERα signals negatively-regulate the PCa invasion. CAF.ERα(-) or ERα(+) cells were treated with vehicle, E2 (10 nM) or/and ICI182,780 (10 μM) and co-cultured with macrophages for 48 hr. CMs were collected and added to 24-well plates and the PCa cells (C4-2) were seeded into inserted transwells pre-coated with matrigel. After 48 hr of incubation, invaded PCa cells were counted and compared, and quantitation data is shown below the images. [file 12943_2015_488_MOESM1_ESM.pdf]

Yeh *et al.*, supplemental Fig. 2.

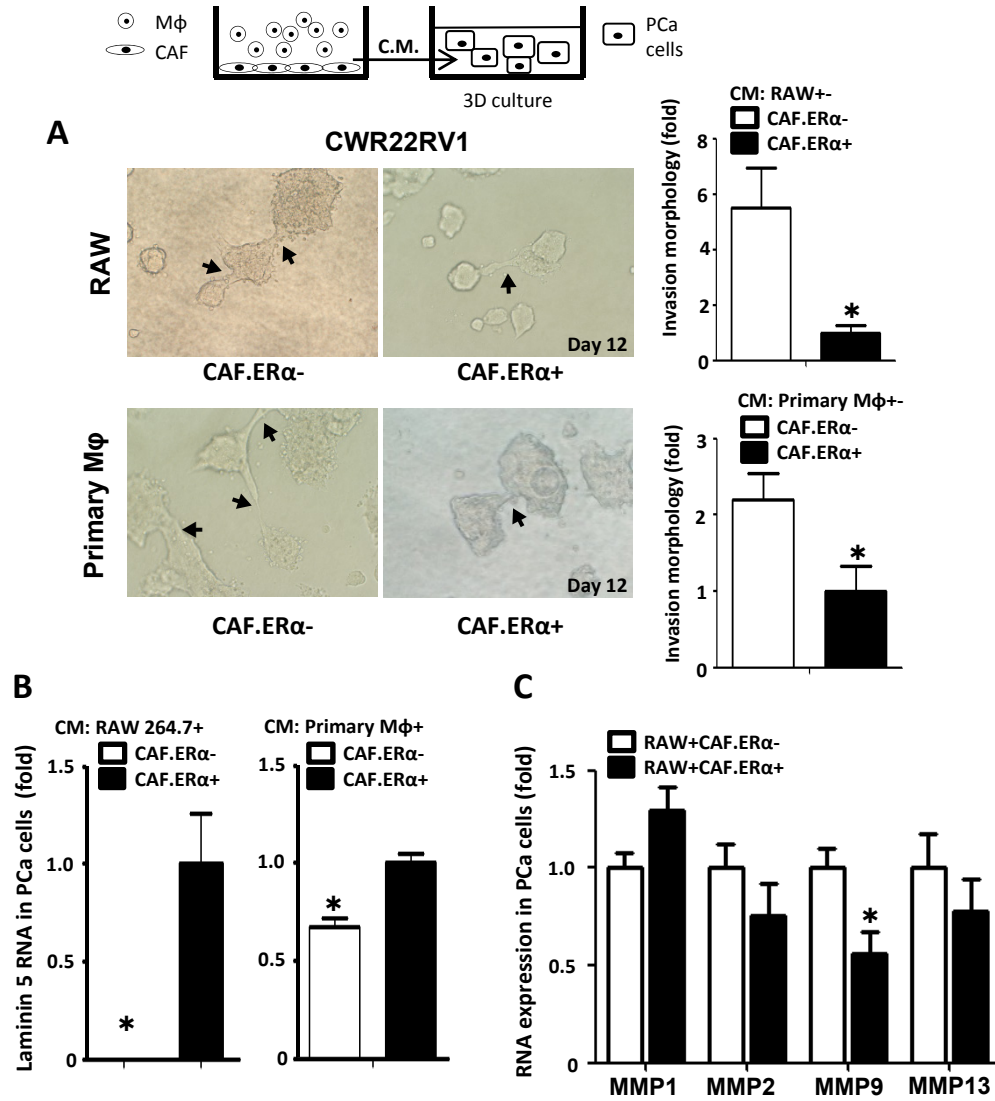

Supplement: Additional file 2: Figure S2. — CM from co-cultured CAF/macrophages affects PCa invasion in the 3D culture system. The carton illustrates the experimental system. CM was collected from co-culture of CAF.ERα(+) or CAF.ERα(-) cells and RAW264.7 cells or B6 primary macrophages (Mφ) for 24 hr. The CM was then used to treat CWR22Rv-1 cells for 3 days, then seeded in 3D environment for 12 days to form inter-acinar bridges. (A) Numbers of inter-aciniar bridges were counted per field and quantifications are in right panels. (B) Laminin 5 mRNA was quantified to show the invasive potential of the PCa cells in the 3D culture environment. At Day 12, RNA was extracted from PCa cells and expression levels of laminin 5 were measured by qPCR and quantifications are shown. (C) Expression of invasion related marker, MMP9, was also demonstrated by Q-PCR in CWR22Rv-1 cells pre-inoculated with the collected CM, quantification is shown. *, P < 0.05 vs. CM from CAF.ERα(-)/macrophage group. [file 12943_2015_488_MOESM2_ESM.pdf]

Yeh *et al.*, Supplementary Fig. 3.

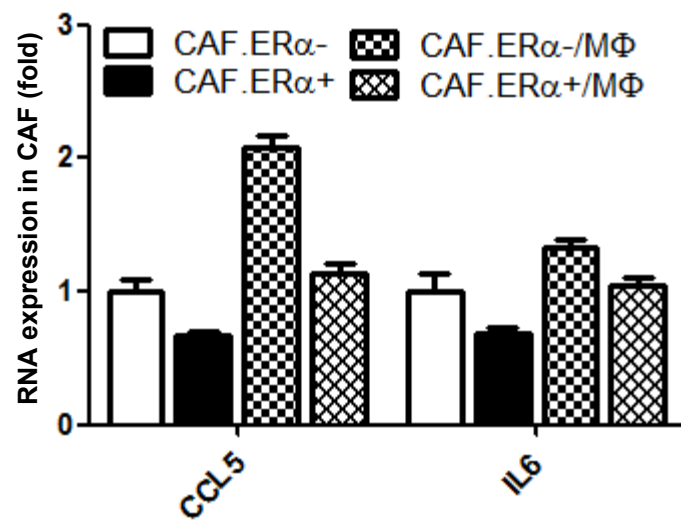

Supplement: Additional file 3: Figure S3. — Infiltrated Macrophages (Mφ) can affect the macrophages recruited-related gene profiles in CAF cells. CAF.ERα(-) or CAF.ERα(+) cells were co-cultured with macrophages for 2 days. We compared gene profiles of macrophages attraction related CCL5 and IL6 in CAF.ERα(+) and CAF.ERα(-) using qPCR. [file 12943_2015_488_MOESM3_ESM.pdf]

Yeh *et al.*, Supplementary Fig. 4.

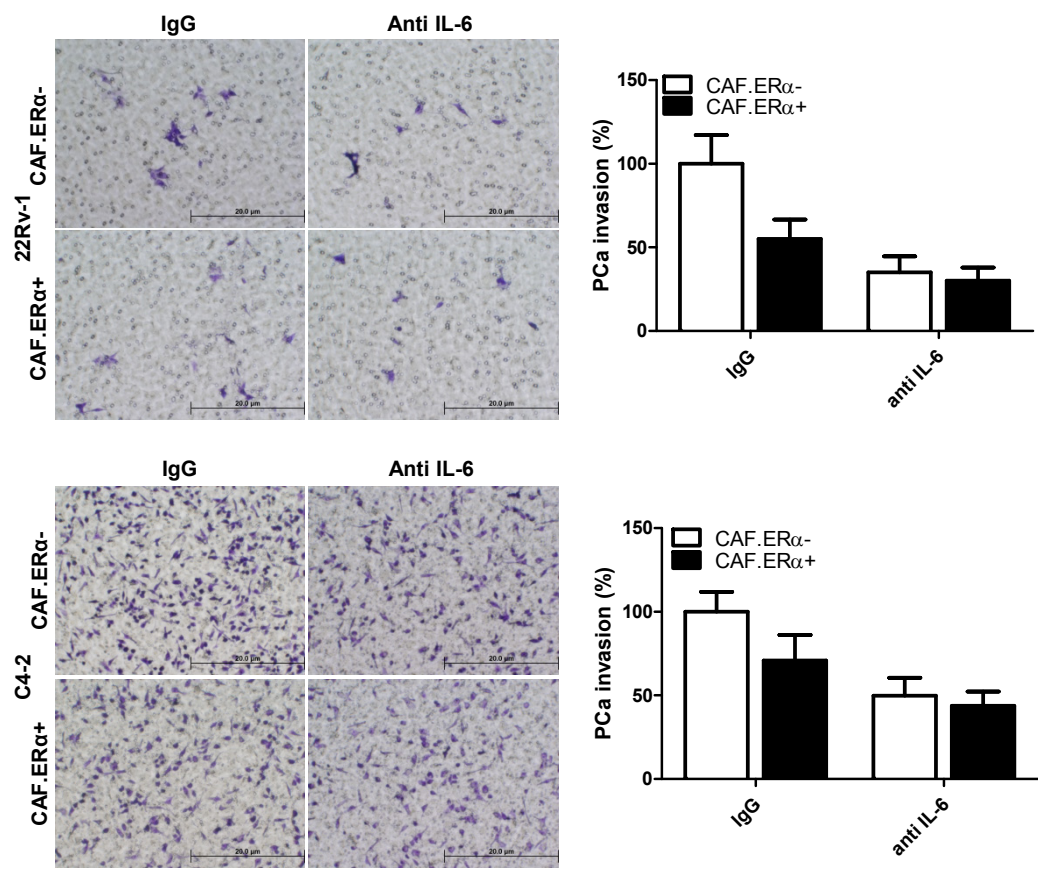

Supplement: Additional file 4: Figure S4. — Co-culture of CAF ERα(+) cells and PCa cells can decrease PCa cell invasion through changing IL-6 expression. CM was collected from CAF.ERα(-) and CAF.ERα(+) cells together with or without with IL-6 neutralizing antibody (Anti IL-6) or IgG (control) into bottom wells of 24-well transwell systems. We then trypsinized and seeded CWR22Rv-1 and C4-2 cells (1x10^5^) into matrigel-pre-coated transwells for invasion assay. Quantitation is at right. [file 12943_2015_488_MOESM4_ESM.pdf]

Yeh *et al.*, Supplementary Fig. 5.

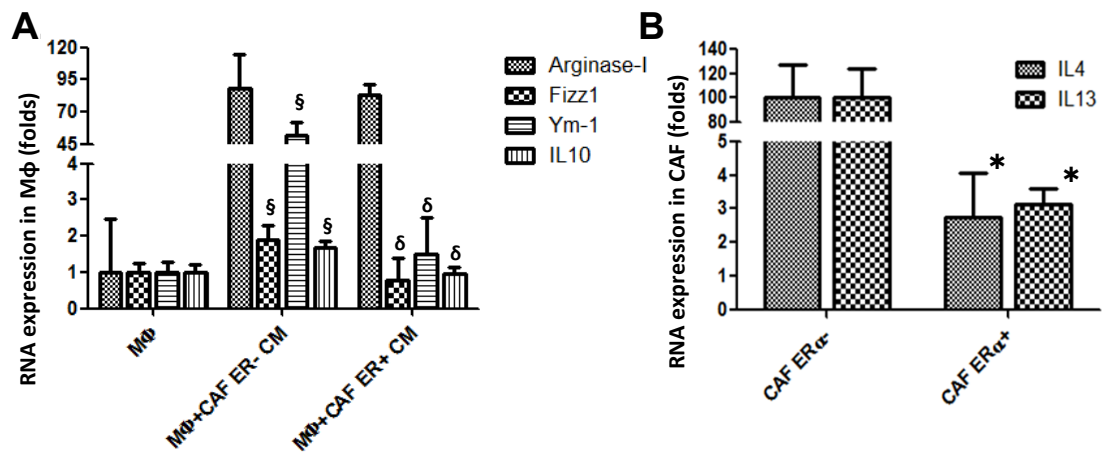

Supplement: Additional file 5: Figure S5. — CAF.ERα(+) can reduce the M2 marker expressions in the co-cultured macrophages (Mφ). (A) ERα in CAF cells inhibits M2-type macrophages transformation. (B) mRNA expressions of IL-4 and IL-13 in CAF cells were assayed by qPCR. mRNA expressions of M2 markers in macrophages were assayed by qPCR. After incubating with CM collected from CAF.ERα(+) or CAFERα(-), macrophages were collected to detect M2 markers by qPCR. *, P < 0.05 vs. CAF.ERα(-) cells; §, P < 0.05 vs. Mφ; δ, p < 0.05 vs. Mφ/CAF.ERα(-) CM. [file 12943_2015_488_MOESM5_ESM.pdf]
